# A Cognitive Training Programme on Cancer-Related Cognitive Impairment (CRCI) in Breast Cancer Patients Undergoing Active Treatment: A RCT Study Protocol

**DOI:** 10.3390/jcm14145047

**Published:** 2025-07-16

**Authors:** Samuel Jiménez Sánchez, Celia Sánchez Gómez, Susana Sáez Gutiérrez, Sara Jiménez García-Tizón, Juan Luis Sánchez González, María Isabel Rihuete Galve, Emilio Fonseca Sánchez, Eduardo José Fernández Rodríguez

**Affiliations:** 1 Department of Social Psychology and Anthropology, University of Salamanca, 37007 Salamanca, Spain; samueljs@usal.es; 2 Department of Developmental and Educational Psychology, University of Salamanca, 37007 Salamanca, Spain; sarajim@usal.es; 3 Institute of Biomedical Research of Salamanca (IBSAL), 37007 Salamanca, Spain; susanasg@usal.es (S.S.G.); juanluissanchez@usal.es (J.L.S.G.); rihuete@usal.es (M.I.R.G.); edujfr@usal.es (E.J.F.R.); 4 Department of Nursing and Physiotherapy, University of Salamanca, 37007 Salamanca, Spain; 5 Department of Medical Oncology, University Hospital of Salamanca, 37007 Salamanca, Spain; efonseca@usal.es

**Keywords:** breast cancer, cancer-related cognitive impairment, cognitive training, everyday cognition, randomised controlled trial

## Abstract

**Background:** In light of increasing breast cancer survival rates, it is essential to address cancer-related cognitive impairment (CRCI), a common yet often underestimated symptom. **Methods:** A randomised controlled trial is proposed involving 50 newly diagnosed participants, divided into a control group (CG) and an intervention group (IG). Both groups will receive an educational leaflet, while the IG will also take part in an individualised cognitive training programme based on everyday cognition (80 sessions distributed across four periods, compiled in a training dossier). Cognitive, emotional, and functional variables will be assessed before and after the intervention: cognitive function (MoCA test), everyday cognition (PECC), anxiety (Hamilton), functionality (LB), sleep quality (PSQI), quality of life (ECOG), and subjective memory complaints (FACT-COG). **Expected results:** Findings may guide future interventions and tailored protocols to alleviate CRCI in breast cancer patients undergoing active treatment. **Ethics and dissemination:** This study was approved by the Ethics Committee of the University of Salamanca (PI 2023 12 1478-TD).

## 1. Introduction

Breast cancer is one of the most common neoplasms worldwide and represents a leading cause of cancer-related morbidity among women. In Spain, 35,001 new cases were diagnosed in 2023, making it the most frequent cancer among women, according to the Sociedad Española de Oncología Médica (SEOM) [1]. The development of early detection strategies and continuous improvements in treatment have significantly increased survival rates in recent decades [2]. However, these advances have also led to growing recognition of adverse effects that impact long-term quality of life, particularly those resulting from adjuvant therapies [3,4,5,6,7].

This epidemiological shift highlights the need to address sequelae arising from both the disease and its treatments, notably cancer-related cognitive impairment (CRCI) [3]. CRCI is defined as a set of cognitive dysfunctions that may occur before, during, or after oncological treatment, including chemotherapy, hormone therapy, and immunotherapy [8,9]. Its prevalence among breast cancer patients varies across studies, but it is estimated that between 17% and 75% of patients undergoing treatment experience some degree of cognitive impairment. This impairment may persist or even worsen in approximately 60% of cases in the long term [10,11,12]. The most affected domains include attention, memory, processing speed, and executive functions, leading to significant difficulties in planning, organising, and performing daily activities [8,9].

Beyond the neuropsychological impact, CRCI has become a central clinical concern due to its effects on patients’ everyday functionality and their social and occupational reintegration. Many women face barriers in resuming their pre-treatment routines, which can negatively affect their self-esteem, independence, and interpersonal relationships. Furthermore, this impairment often coexists with other physical and emotional sequelae of cancer, such as chronic fatigue or depressive symptoms, creating a complex clinical picture that requires an interdisciplinary approach [13]. For this reason, it is crucial to use sensitive tools that allow for the identification and treatment of cognitive difficulties in real-world settings, beyond the controlled environment of traditional testing.

The impact of CRCI, therefore, transcends the neuropsychological domain, affecting patients’ functional ability and quality of life by hindering the performance of instrumental activities of daily living that require the complex integration of cognitive processes—a concept known as “everyday cognition” [14]. Everyday cognition refers to the ability to carry out habitual tasks that demand cognitive skills applied in real and meaningful contexts, such as managing money, preparing meals, or handling administrative matters. Assessing this dimension is essential for understanding the extent to which cognitive impairment affects the autonomy and functional performance of breast cancer patients.

Despite increasing documentation of CRCI, empirical evidence on its functional impact in daily life remains limited [15]. A particularly complex issue is the discrepancy between self-reported cognitive complaints and deficits identified through objective neuropsychological testing [16], a phenomenon also observed in other populations, such as older adults [17]. This incongruence, along with variability in the domains assessed, the lack of standardised instruments, and methodological heterogeneity between studies, hampers the formulation of robust conclusions [9].

In this context, focusing assessment on everyday cognition emerges as a more methodologically appropriate and clinically relevant strategy for evaluating the functional impact of cancer-related cognitive impairment (CRCI). Unlike standardised neuropsychological tests, which evaluate cognitive functions in isolation, everyday cognition more sensitively captures the difficulties patients face in their daily functioning. This is particularly valuable in identifying impairments that significantly compromise their autonomy.

Nevertheless, interventions commonly used to address CRCI still rely on traditional cognitive stimulation programmes. These interventions focus on repetitive, structured tasks aimed at improving specific domains such as attention, memory, or processing speed. While some studies have demonstrated improvements in cognitive performance, the transfer of these gains to real-life contexts has been limited.

To address these limitations, intervention programmes based on everyday cognition represent an innovative alternative with greater potential to produce sustainable and clinically meaningful changes. These programmes integrate principles of neuroplasticity and cognitive training into real or simulated activities that reflect the functional demands of patients’ usual environments. This approach not only enhances cognitive performance but also promotes collateral benefits in emotional and functional well-being [18].

The proposed intervention is a structured cognitive training programme based on the principles of everyday cognition, designed to address the specific functional needs of women undergoing active treatment for breast cancer. The training consists of 80 sessions distributed over four months, delivered through a printed workbook that includes activities closely mirroring instrumental activities of daily living (IADLs). These tasks involve situations such as managing medications, navigating transport, planning meals, or handling financial and administrative duties—activities that demand the integration of multiple cognitive functions, including attention, reasoning, memory, and executive control. By simulating these real-world scenarios, the intervention seeks to improve not only isolated cognitive domains but also patients’ ability to function independently in their daily environments. The use of a hybrid follow-up model (in-person and remote) and a flexible pacing structure further enhances accessibility and adherence. This functional, ecologically valid approach aligns with current recommendations for personalised, context-sensitive rehabilitation in oncology and provides a clinically relevant alternative to traditional cognitive training programmes with limited real-life transfer.

The proposed activities, originally developed for older adults, are centred on basic and instrumental activities of daily living (ADLs and IADLs), given their relevance in assessing real-life cognitive functioning. Although many patients with cancer-related cognitive impairment (CRCI) do not meet clinical thresholds in objective neuropsychological testing, they frequently report subjective cognitive complaints that significantly interfere with daily functioning. In this population, especially among women undergoing active treatment for breast cancer, subtle deficits in attention, working memory, and executive function can impair the performance of everyday tasks—even in the absence of clinically diagnosed cognitive decline. Therefore, implementing interventions focused on functional domains is justified, as they target ecologically valid difficulties that may not be fully captured by conventional cognitive assessments but have a tangible impact on autonomy and quality of life.

Therefore, we propose the following study objectives. The primary objective is to evaluate the effectiveness of implementing a cognitive training programme based on everyday cognition to mitigate the effects of cancer-related cognitive impairment (CRCI) in newly diagnosed breast cancer patients.

The secondary objectives are as follows:

- To describe the presence of cancer-related cognitive impairment (CRCI) in breast cancer patients undergoing active treatment.
- To analyse the influence of anxiety symptoms on cancer-related cognitive impairment (CRCI) in this population.
- To analyse the impact of sleep quality on CRCI in breast cancer patients undergoing active treatment.
- To describe the influence of age on CRCI in patients receiving treatment.
- To assess the impact of chemotherapy treatments on CRCI in this population.

## 2. Materials and Methods

### 2.1. Study Design and Setting

A randomised, parallel-group, controlled clinical trial will be conducted, featuring two fixed-assignment arms: an experimental group and a control group. The design adheres to the Consolidated Standards of Reporting Trials (CONSORT) guidelines [19]. This study will span one year and will be carried out at the University of Salamanca (Spain), specifically within the Oncology Department of the University Healthcare Complex of Salamanca (CAUSA). The current treatment protocol is described in accordance with the SPIRIT recommendations [20] (Figure S1, SPIRIT Figure). The protocol has received approval from the Research Ethics Committee on Medicines of the Salamanca Health Area (Ethics Code: CEIm PI 2023 12 1478—TD) (Supplementary Materials, Favourable report) and complies with the principles of the Declaration of Helsinki. The trial has been registered on ClinicalTrials.gov (registration number NCT06686823) (Supplementary Materials, Study flow diagram).

### 2.2. Participants and Eligibility Criteria

Participants will be recruited through the Medical Oncology Department at the University Healthcare Complex of Salamanca. Adults (18 years or older) visiting the Oncology Department will be contacted by telephone, provided they do not have a clinical diagnosis of a neurological disorder listed in the DSM-5 at the time of inclusion. Upon admission, participants will be given an informative document explaining the present study and necessary details for full understanding.

Participant inclusion will follow a consecutive sampling method, based on the following selection criteria:

Inclusion criteria:

- Age of 18 years or older.
- Recent histopathological diagnosis of breast cancer and initiation of oncological treatment.
- Willingness to voluntarily participate in the present study and sign the informed consent form.

Exclusion criteria:

- Illiteracy or significant deficits in language comprehension.
- Diagnosis of a central nervous system tumour and participation in another cognitive stimulation programme.
- Clinical diagnosis of a neurocognitive disorder listed in the DSM-5.

Withdrawal Criteria:

- Withdrawal from the programme.
- Failure to complete the final assessment.

### 2.3. Intervention

Two parallel intervention programmes will be designed, corresponding to the two study groups: a Health Education Programme (for both the control group [CG] and the intervention group [IG]) and a Cognitive Training Programme (CT) exclusively for the intervention group, as detailed below following the TIDieR (Template for Intervention Description and Replication) guidelines [21]. As can be seen in Table 1 and Table 2.

#### 2.3.1. Intervention Group

The cognitive training (CT) programme in everyday cognition (EC) will be administered individually. Each participant will be provided with a dossier specifically developed for this study. All participants receive the same tasks in the dossier with no adaptation according to level of difficulty or functional profile. This will be carried out in parallel to the Health Education Programme, which, as mentioned above, will be carried out in both study groups (control and intervention).

The intervention comprises four training periods, each lasting one month (Periods 1 to 4: P1–P4), with 20 activities per period. Participants will complete five activities per week across each month.

To ensure adherence and therapeutic compliance, two types of follow-ups will be conducted: in-person and remote (via video call).

At the end of P1 and P3 (months 1 and 3), follow-up will be conducted remotely.

At the end of P2 and P4 (months 2 and 4), follow-up will be in person, involving an in-clinic consultation to assess progress and address any issues.

The final evaluation (FE) will coincide with the end of P4.

The intervention will use materials resembling those encountered in real-life tasks or in the resolution of everyday problems, with the ultimate goal of aligning the intervention with the participants’ daily lives. The activities will require the use of cognitive functions such as attention, reasoning, working memory, planning, and processing speed in the context of instrumental activities of daily living, including the following: medication adherence, meal preparation, housekeeping, transport use, telephone use, financial management, and accessing information and current events. These tasks include contextual and meaningful elements, as they are drawn from real-life scenarios that patients must navigate to maintain autonomy and independence in society.

Currently, there is limited information in Spain regarding training in everyday cognition. The training dossier was developed based on the study by Sánchez and Fernández [22] and Sáez-Gutiérrez et al. [23], which includes practical examples of session formats.

For example, one everyday cognition training activity focuses on medication adherence. A practical case is presented where “Marisa” (a fictional person) visits her doctor and is given a prescription leaflet. She must read and comprehend the leaflet to answer a set of questions. In a second task, participants are required to read, understand, and memorise a medication sheet and answer questions without referring back to the material.

#### 2.3.2. Control Group

The control group will receive an informational leaflet with instructions and recommendations for maintaining an active and healthy lifestyle, promoting self-care, and practising good health practices. The leaflet will include updated WHO guidelines with specific recommendations to reduce the risk of cognitive decline. These include eating healthy foods, engaging in physical activity, maintaining social contact, doing challenging cognitive games, sleeping well, managing stress, staying hydrated, and avoiding smoking and excessive alcohol consumption.

**Figure 1:**
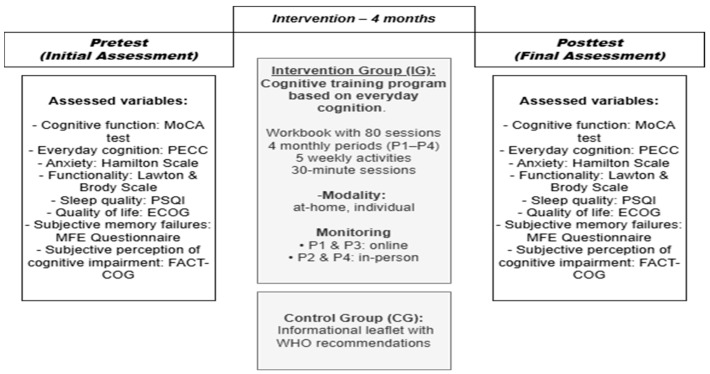
Checklist for the description and replication of interventions (TIDieR) in the control group.

In summary, two parallel intervention programmes will be implemented, corresponding to the two study groups: a Health Education Programme, provided to both the control group (CG) and the intervention group (IG), and a Cognitive Training Programme (CT), administered exclusively to the intervention group (IG), over a period of four months, with pre- and post-intervention assessments. As can be seen in Figure 1.

### 2.4. Outcomes

In the baseline assessment, all variables will be measured, including sociodemographic factors. Subsequently, all outcome variables will be reassessed following the completion of the four-month intervention.

The personal and sociodemographic variables include age, weight, height, body mass index, time since diagnosis, marital status, treatment, and caregiver status.

#### 2.4.1. Primary Outcomes

The primary outcome will be cognitive function, assessed using the Montreal Cognitive Assessment Test (MoCA) and subjective cognitive perception using the FACT-Cog scale.

“Montreal Cognitive Assessment Test (MoCA Test), Version 8.3” [24]: This tool detects mild cognitive impairment (MCI) by assessing executive functions, attention, abstraction, memory, calculation, orientation, etc. Administration time is approximately 10 min. The maximum score is 30 points, with scores below 26 indicating possible MCI (in developed countries).

“FACT-Cog”: Functional Assessment of Cancer Therapy—Cognitive Function (Version 3) [25]: This 37-item questionnaire is divided into six cognitive domains: memory, concentration, mental acuity, verbal fluency, functional interference, and multitasking ability. It also includes two subscales: “perceived comments from others” and “impact of perceived cognitive impairment on quality of life”. Respondents use a 5-point Likert scale ranging from 0 (“never”) to 4 (“several times a day”) to rate occurrences over the past seven days. Subscale scores are summed to yield a total FACT-Cog score (0–148), with higher scores indicating better cognitive function.

#### 2.4.2. Secondary Outcomes

The secondary outcomes are as follows:

Everyday Cognition: “Test for the Assessment of Everyday Cognition (PECC)” [26]: Assesses the ability to resolve 12 real-life situations in the areas of medication, administrative management, financial management, meal preparation, transport, and shopping. It provides insight into functional capacity in daily life. Administration time is approximately 35 min.

Anxiety: “Hamilton Anxiety Rating Scale” [27]: A clinical evaluation tool used to assess the level of anxiety experienced. It consists of 14 items, each with five response options ranging from “not present” to “very severe”. Final scores categorise anxiety levels as follows: ≤17 (mild), 18–24 (moderate), and 25–30 (severe).

Functionality: “Lawton and Brody Instrumental Activities of Daily Living Scale” [28]: Designed to assess autonomy in instrumental activities among older adults. It is scored on a binary scale (0 or 1) for each item, with a total score of 8. Administration time is approximately 4 min.

Sleep Quality: “Pittsburgh Sleep Quality Index (PSQI)” [29]: Developed to assess sleep quality in individuals aged 24 to 83. It includes 19 items grouped into seven components: subjective sleep quality, sleep latency, sleep duration, habitual sleep efficiency, sleep disturbances, use of sleep medication, and daytime dysfunction. Administration time is 5–10 min.

Quality of Life: “ECOG Performance Status Scale” [30]: A single-item measure used to assess the impact of illness on the patient’s quality of life. It offers five possible performance status scores.

Subjective Memory Complaints: “Everyday Memory Failures Questionnaire” [31]: Categorises memory lapses under “speaking, reading, and writing”, “names and faces”, “actions”, and “learning new things”. Responses are rated using a 9-point Likert scale ranging from “Not once in the last 3 months” to “More than once a day”. Given the complexity of the 9-option scale, some authors have used fewer response options.

### 2.5. Sample Size

The sample size estimation is based on the expected outcomes of the primary variable: changes in symptoms of cognitive impairment (MoCA score). The reference is a study with similar characteristics conducted in a female population without diagnosed cognitive impairment by Park and colleagues [32]. That study aimed to assess the effect of a cognitive stimulation programme on improving cognitive abilities in older adults without cognitive decline. A pretest–posttest design was used, with the intervention delivered once weekly over three months.

In that study, the MoCA score increased by 2.95 points. Based on these parameters and accepting an alpha risk of 0.05 and a beta risk below 0.2 in a two-tailed test, 25 subjects per group (intervention and control) are required to detect a difference of 2.95 points or more. A common standard deviation of 3.48 is assumed. A 10% attrition rate has been estimated.

Inclusion and Randomisation Procedure:

If an individual agrees to participate in the study, an in-person consultation will be scheduled. One of the study researchers will present the study, clarify any doubts, and obtain written informed consent. After confirming that the subject meets the inclusion criteria, a baseline evaluation will be conducted to collect clinical, sociodemographic, and study outcome variables. Finally, participants will be randomly assigned to one of the two groups (IG—cognitive training group or CG—control group). Randomisation will be performed using the Epidat 4.2 software with a 1:1 allocation ratio. Group allocation will remain concealed until assignment. A final assessment will be carried out for all participants four months after randomisation (coinciding with the end of the first phase of oncological pharmacological treatment), reassessing clinical, sociodemographic, and outcome variables.

### 2.6. Assignment and Randomisation

Sequence generation, randomisation, participant recruitment, and group allocation will be conducted by research staff not involved in the evaluation or delivery of interventions. This approach is intended to reduce potential bias of the present study.

### 2.7. Blinding

Participants will be blinded to their group assignment and unaware of the specific intervention they will receive. To minimise potential contamination between groups, external research staff—trained beforehand—will be responsible for conducting the assessments. These assessors will be blinded to group assignments to ensure objective evaluation. The clinical trial will therefore include a third-party blinded outcome assessment. Additionally, researchers in charge of the statistical analysis will also be blinded to enhance the rigour and scientific validity of this study.

Participants will be assessed at two timepoints during this study: the initial assessment (baseline) and the final assessment (FA) following the intervention. The baseline evaluation will occur after recruitment and before group allocation, recording all study variables and administering the planned objective tests. Following randomisation, each group will undergo its assigned intervention. Afterwards, the final assessment will be conducted, using the same tests as at baseline. This final assessment will take place four months after the initial one, matching the duration of the oncological treatment regimen.

Participants who request it at enrolment will receive a personalised report of their results and progress. All evaluations will be conducted by trained and qualified research personnel.

### 2.8. Statistical Methods

To analyse the data descriptively, normality will be assessed using the Kolmogorov–Smirnov and Shapiro–Wilk tests. Variables that follow a normal distribution will be described using the mean, standard deviation, and range. For variables that do not follow a normal distribution, the median and interquartile range will be used. Categorical variables will be presented as frequencies and percentages.

In the quantitative analysis, correlations will be examined to validate the assessment tools used in this study, applying Pearson’s correlation coefficient for normally distributed data. To assess reliability, Cronbach’s alpha coefficient will be calculated. For comparing two means, Student’s *t*-test will be used in cases where parametric assumptions are met, while the Mann–Whitney U test and Wilcoxon signed-rank test will be employed for non-parametric data, including repeated measures.

When comparing three or more means, an ANOVA will be conducted for parametric data using Snedecor’s F test, whereas the Kruskal–Wallis H test will be used for non-parametric data. For repeated measures, the F test and the Friedman test will be applied for parametric and non-parametric data, respectively. Depending on the distribution, either Pearson’s or Spearman’s correlation tests will be used to assess relationships between variables.

A multivariate logistic regression analysis will be performed to identify variables associated with events of interest. Variables that are statistically significant in the bivariate analysis or deemed clinically relevant will be included in the model. Categorical variables will be analysed using contingency tables and the Chi-square test, where appropriate. Statistical significance will be established at *p* < 0.05, corresponding to a 95% confidence interval.

In addition to statistical significance, the magnitude of the effect or improvement will be estimated to determine the clinical relevance of the results. Effect sizes will be calculated using Cohen’s d for differences between means and r or η^2^ (eta squared) for correlations and variance analyses, respectively. For categorical outcomes, odds ratios (ORs) with their corresponding confidence intervals will be reported. These measures will help interpret the practical significance of the observed differences and associations, providing a more comprehensive understanding of the impact of the intervention.

In order to enhance transparency and replicability, the statistical analysis will specify the role of each variable within the models. The primary dependent variables will be cognitive performance scores (MoCA and FACT-Cog), while the main independent variable will be group allocation (intervention vs. control). Secondary outcomes—such as everyday cognition (PECC), anxiety (Hamilton), sleep quality (PSQI), and functionality (Lawton and Brody)—will be analysed as both dependent and potential covariates depending on the model. Multivariate analyses will include sociodemographic variables (e.g., age, educational level) and clinical variables (e.g., treatment type) as covariates to control for their potential confounding effects. A detailed mapping of statistical tests to each outcome variable will be included in Supplementary Materials to facilitate replication.

All analyses will be performed using IBM SPSS Statistics software, version 28.0.1.

## 3. Discussion

### 3.1. Potential Impact and Relevance of This Study

Cancer-related cognitive impairment (CRCI) is one of the most common adverse effects experienced by women with breast cancer, affecting key cognitive functions such as memory, attention, and processing speed. In this context, an intervention based on everyday cognition represents a promising strategy, as it focuses on practical skills used in daily life and therefore holds significant ecological transfer potential. It is expected that this type of training will lead to meaningful improvements in overall cognitive functioning, particularly in working memory [33], processing speed [34], executive functions, and prospective memory [35], positively influencing the performance of daily living activities.

Additionally, patients are expected to perceive subjective improvements in their cognitive abilities, which can be assessed using instruments such as the FACT-Cog. In this regard, notable changes may be observed in perceived cognitive impairment (PCI) and perceived cognitive abilities (PCA), with potential benefits for emotional well-being and everyday functional performance.

Nonetheless, results must be interpreted with caution, as treatment implementation varies widely across the literature. Some interventions are delivered digitally [29,35,36,37,38,39,40], while others use paper-based formats [30], with substantial differences in duration and number of sessions. In the present protocol, a manual intervention using a workbook designed for everyday cognition training is proposed, tailored to the functional context of the patients.

While digital cognitive training programmes have demonstrated promising results, they often require access to specific devices, stable internet connections, and a certain level of digital literacy, which may limit their applicability in some clinical contexts. In contrast, paper-based interventions such as the one proposed here offer several practical advantages: they are more cost-effective, easier to implement in routine care settings, and accessible to a broader range of patients, including those with limited technological resources or skills. Moreover, paper-based formats may enhance adherence by reducing cognitive and technological barriers, particularly in patients experiencing fatigue, anxiety, or treatment-related distress. Although scalability may appear more limited compared to digital platforms, the manual format allows for greater contextual adaptation and personalisation of support, which can be critical for promoting engagement and functional transfer. Future studies should explore direct comparisons between both modalities, but the present choice reflects a strategic decision to maximise feasibility, accessibility, and ecological validity in a real-world oncology setting.

Moreover, the inclusion of emotional variables such as anxiety and depression is emphasised due to their direct influence on cognitive performance [41]. This is especially relevant for women with breast cancer, a population often experiencing elevated emotional distress [32]. Improving emotional health may in turn enhance cognitive recovery, creating a mutually reinforcing relationship.

The proposed intervention protocol is an innovative tool aimed at women undergoing active treatment for breast cancer, with the goal of mitigating CRCI from the early stages of the oncological process. Unlike previous research focusing on post-treatment survivors, this proposal takes a preventive approach, which could offer significant clinical benefits. For example, a recent study [42] evaluating web-based cognitive training in breast cancer survivors found no significant improvements in working memory or perceived cognitive functioning, although benefits were reported in verbal learning and working memory at five months. This protocol aims to address such limitations through a longer (four months long) and more contextually grounded intervention, designed to support real and lasting skill transfer.

In alignment with this, studies using adaptive cognitive training have shown both objective improvements (e.g., in working memory capacity and cognitive efficiency) and subjective benefits (e.g., improved perceived cognitive ability and reduced depressive symptoms), with effects sustained up to one year post-intervention [33,43]. The present protocol is based on similar adaptive principles but integrates tasks directly linked to patients’ everyday environments (e.g., medication adherence, household task management, or public transport use), potentially further enhancing the consolidation of meaningful and functional learning.

Furthermore, the literature suggests that not only the type of intervention but also its timing, duration, intensity, and practical applicability in everyday life are critical factors for effectiveness. In this regard, studies such as that by Sousa et al. reported no significant differences between groups receiving structured attention training or compensatory strategies and the control group [38], underscoring the importance of a functional approach, as proposed here. This model stands out due to its extended structure, hybrid follow-up (in-person and telematic), and inclusion of real-life activities, all of which may improve adherence and treatment effectiveness.

A recent systematic review [44] concluded that cognitive training can improve domains such as verbal memory and working memory, although no consistent effects were found for attention, executive function, or emotional symptoms. This highlights the need for broader and more contextualised interventions. In response, the current protocol proposes training that spans multiple cognitive domains (attention, reasoning, processing speed, and planning), integrating them into functional daily-life tasks.

From a qualitative perspective, programmes such as BrainHQ have been found to be engaging and satisfactory, although barriers such as perceived failure or fatigue have also been reported [30]. Therefore, the current study is designed with a flexible structure, adapted to each patient’s pace and supported through regular follow-ups, which may help reduce frustration and dropout, thereby improving acceptability and adherence.

In sum, this clinical protocol offers an innovative intervention model that integrates insights from the existing scientific literature while incorporating significant methodological improvements: functional contextualisation of training, hybrid follow-up, sufficient programme duration, and comprehensive evaluation. If proven effective, this model could become an accessible, sustainable, and clinically applicable tool for addressing CRCI from the early stages of oncological treatment.

In addition to assessing the efficacy of the intervention, the present study also aims to provide a comprehensive characterisation of cancer-related cognitive impairment (CRCI) in women undergoing active treatment for breast cancer. Although CRCI has been widely documented in survivorship stages, less is known about its manifestation during treatment, particularly in terms of its prevalence and functional consequences. Describing the cognitive profile of this population during active treatment will help identify the most affected domains and inform timely intervention strategies, ultimately contributing to improved clinical decision-making and patient care.

Furthermore, thi study examines several variables that may modulate CRCI. Anxiety symptoms are known to interfere with attentional control and working memory, potentially exacerbating cognitive complaints. Likewise, poor sleep quality has been strongly associated with reduced cognitive performance, particularly in domains such as attention, processing speed, and executive function. By analysing these factors alongside age and type of treatment (e.g., chemotherapy), the protocol seeks to better understand individual differences in CRCI. Such insights may allow for the identification of high-risk subgroups and the development of more personalised cognitive support strategies, enhancing both prevention and management efforts throughout the cancer care continuum.

### 3.2. Limitations

Although this study has been designed with a robust methodological approach, several relevant limitations must be considered. First, this study is being conducted at a single hospital centre, which may limit the representativeness and generalisability of the findings to populations with different clinical, geographical, or sociocultural characteristics. This limitation should be considered when interpreting the results.

Second, the absence of longitudinal follow-up beyond the four-month intervention period prevents assessment of the long-term sustainability of the cognitive training effects.

Additionally, the potential presence of clinical comorbidities, whether pre-existing or treatment-induced, may influence intervention response and the stability of cognitive function, affecting the sample’s homogeneity. The fact that the same professional may conduct both the intervention and the evaluation introduces a risk of observer bias, which could affect the objectivity and validity of the findings. These limitations should be taken into account when evaluating the applicability and robustness of the results.

### 3.3. Ethics and Dissemination

This study received favourable ethical approval from the Research Ethics Committee on Medicines of the Salamanca Health Area on 11 March 2024 (PI 2023 12 1478-TD). All participants will sign informed consent forms in accordance with the principles of the Declaration of Helsinki. Participants will be informed about the study objectives and the risks and benefits of the procedures involved. None of the procedures pose life-threatening risks for the type of subjects to be included in this study.

As this study involves the collection of biological samples, participants will be thoroughly informed before giving their consent. Throughout the entire process, the confidentiality of participants’ personal data will be safeguarded in accordance with the provisions of Spanish Organic Law 3/2018, of 5 December, on the Protection of Personal Data and Guarantee of Digital Rights, and Regulation (EU) 2016/679 of the European Parliament and of the Council of 27 April 2016 (GDPR), concerning the protection of natural persons with regard to the processing of personal data and the free movement of such data.

## Figures and Tables

**Table 1:** TIDieR checklist for intervention group.

Item No.	Item
Brief Name	
1	Cognitive training programme in everyday cognition.
Why?	
2	To mitigate the effects of cancer-related cognitive impairment (CRCI) in newly diagnosed breast cancer patients.
What?	
3	Materials: A dossier specifically developed for this study. It includes real-life situations that closely resemble daily tasks or problems participants may face, aiming to align the intervention with their everyday experience.
4	Procedures: Participants will use cognitive functions such as attention, reasoning, working memory, planning, and processing speed during instrumental activities of daily living, including medication adherence, meal preparation, housekeeping, use of transport, telephone use, financial management, and access to information and current events.
Who delivers?	
5	All interventions will be conducted by a qualified team member with experience in assisting cancer patients.
How?	
6	The intervention is divided into four monthly training periods (P1–P4), each consisting of 20 activities. The prescription is for the patient to perform 5 activities per week to ensure proper adherence to treatment and to avoid the fatigue effect. To ensure compliance and adherence, follow-up will be both face-to-face and via video call.
Where?	
7	The study, assessments, and intervention will take place at the University Healthcare Complex of Salamanca (CAUSA), the University of Salamanca’s Teaching and Clinical Units (UDATO), and the Salamanca Biomedical Research Institute (IBSAL). Participant recruitment will be conducted through consultations at the CAUSA Day Oncology Unit and the Spanish Association Against Cancer (AECC).
When and How Much?	
8	The intervention will be implemented over four months from the start of this study.
Adaptations	
9	Adaptations will also be available. Due to the diversity of topics and the individual nature of the intervention, sessions will be adapted to each topic. It is not currently possible to evaluate the effectiveness of the therapy.
Modifications	
10	
How well?	
11	Supervision of the therapy will take place through weekly meetings between therapists and researchers.
12	

**Table 2:** TIDieR checklist for control group.

Item No.	Item
Brief Name	
1	Health Education Programme.
Why?	
2	To educate the population on healthy habits to reduce the risk of cognitive impairment.
What?	
3	Materials: An informational leaflet promoting an active and healthy lifestyle, encouraging self-care and best health practices.
4	Procedures: The leaflet includes updated WHO guidelines recommending concrete measures to reduce the risk of cognitive impairment: eating healthy, physical activity, maintaining social contact, cognitive stimulation, good sleep hygiene, stress management, hydration, and avoiding smoking and excessive alcohol.
Who delivers?	
5	All interventions will be conducted by a qualified team member with experience in assisting cancer patients.
How?	
6	The leaflet will be provided individually at the start of the programme. To ensure compliance and adherence, follow-up will be both face-to-face and via video call.
When?	
7	The study, assessments, and intervention will take place at CAUSA, UDATO, and IBSAL. Participant recruitment will occur in consultations at CAUSA’s Oncology Day Unit and at AECC offices.
How much?	
8	The programme will be implemented over four months following study initiation.
9	Adaptations will also be available. Due to the diversity of topics and the individual nature of the intervention, sessions will be adapted to each topic. It is not currently possible to evaluate the effectiveness of the therapy.
Tailoring	
10	
How well?	
11	Supervision of the therapy will take place through weekly meetings between therapists and researchers.
12	

## Data Availability

The original data presented in this study will be openly available in the GREDOS repository of the University of Salamanca.

## Supplementary Materials

The following supporting information can be downloaded at: https://www.mdpi.com/article/10.3390/jcm14145047/s1, Figure S1, SPIRIT Figure; File S1, Favourable report and Study flow diagram.
