# Supplementary material for: A Cognitive Training Programme on Cancer-Related Cognitive Impairment (CRCI) in Breast Cancer Patients Undergoing Active Treatment: A RCT Study Protocol
Source: J Clin Med. 2025 Jul 16;14(14):5047. doi: 10.3390/jcm14145047 (PMC12295130; doi:10.3390/jcm14145047)
Supplement: Supplementary file 1 [file jcm-14-05047-s001.zip › jcm-3739586-supplementary.pdf]

Figure S1. The schedule of enrolment, interventions, and assessments of SPIRIT 2013.

	STUDY PERIOD							
	Enrolment	Allocation	Post-allocation					Close-out
TIMEPOINT	*Month 3*	*Month 4*	*Month 4-6*	*Month 6-8*	*Month 10-12*	*Month 10-12*	*Month 12-14*	*Month 14-16*
**ENROLMENT:**								
**Eligibility screen**	X							
**Informed consent**	X							
Database creation	X							
**Allocation**		X						
**INTERVENTIONS:**								
Intervention A: Experimental group intervention *(Cognitive training program in everyday cognition)*			⟵	⟷	⟷	⟷	⟶	
Intervention B control group intervention (Health Education Programme)			⟵	⟷	⟷	⟷	⟶	
**ASSESSMENTS:**								
Baseline Intervening variables	X							
Outcome variables: Anxiety, sleep quality, functionality, subjective memory loss.	X							X
Dissemination of results								X

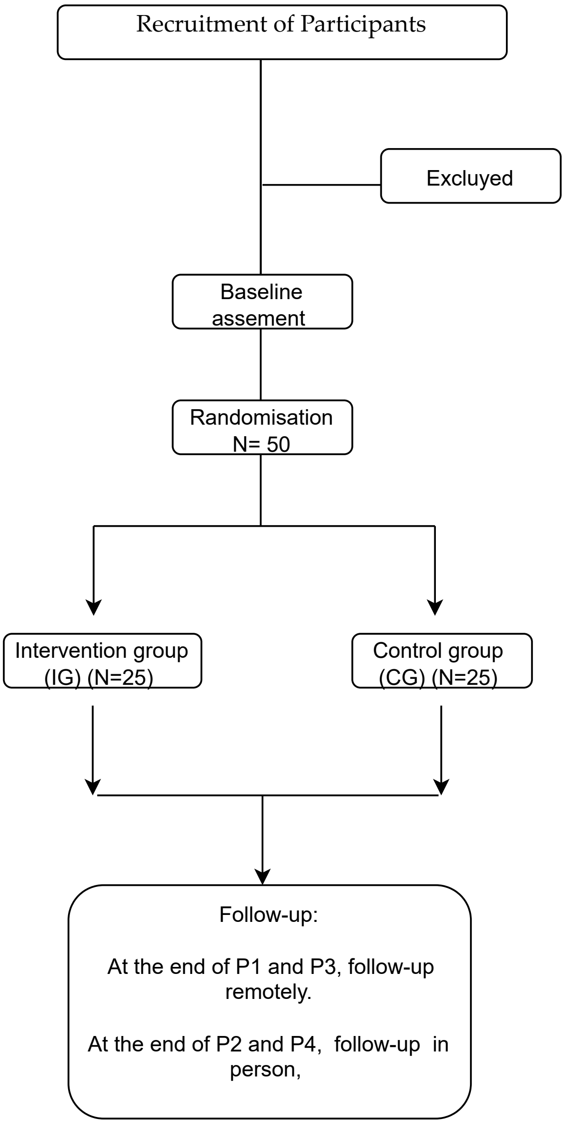
Recruitment of Participants
Excluyed
Baseline assement
Randomisation N= 50
Intervention group (IG) (N=25)
Control group (CG) (N=25)
Follow-up:
At the end of P1 and P3, follow-up remotely.
At the end of P2 and P4, follow-up in person,

Paseo de San Vicente, 58-182 37007
Salamanca
Medicines Research Ethics Committee
Telephone: 923 29 11 00 - Ext. 55 515

E-mail: comite.etico.husa@saludcastillayleon.es

OPINION OF THE COMMITTEE ON THE ETHICS OF RESEARCH INVOLVING MEDICINAL PRODUCTS

Ms. CONCEPCIÓN TURRIÓN GÓMEZ, Technical Secretary of the Salamanca Health Area Research Ethics Committee,

C E R T I F I C A

That this Committee, at its meeting on 22/01/2024 CEIm Ref. 2024/01
has assessed the Research Project entitled

RANDOMISED CONTROLLED TRIAL: EFFECT OF A DAILY COGNITION TRAINING PROGRAMME ON COGNITIVE FUNCTION, EMOTIONAL STATE, QUALITY OF SLEEP AND FUNCTIONING IN BREAST CANCER PATIENTS

CEIm Code: PI **2023 12 1478 - TD**

of which he is Principal Don Samuel Jiménez Sánchez

Investigator of the Oncology

assessed in accordance with Law 14/2007 on Biomedical Research, the ethical principles of the Declaration of Helsinki of the World Medical Association on ethical principles for medical research involving human subjects, as well as the other ethical principles and legal regulations applicable according to the characteristics of the study,
and clarifications resolved

Considers that this study meets the necessary requirements and is feasible to be carried out in this centre, and therefore, it is hereby **APPROVED** for this study to be carried out.

And for the record, this is signed at Salamanca, dated 11 March 2024

TURRION GOMEZ MARIA DE LA CONCEPCION - 07986126C

Digitally signed by TURRION GOMEZ MARIA DE LA CONCEPCION - 07986126C

THE SECRETARIAT

S.D.: Ms. Concepción Turrión Gómez

Composition of the CEIm of the Salamanca Health Area
President: Mr. Enrique Nieto Manibardo (CAUSA Data Protection Delegate) Vice-president:
Ms. Teresa Martín Gómez (Specialist in Oncology)
Secretary: Ms. Concepción Turrión Gómez (Pharmacist and Biochemist - Representative of the Scientific Committee - IBSAL). Members: Mr. Ricardo Tostado Menéndez (Clinical Pharmacologist); Ms. Silvia Jiménez Cabrera (Hospital Pharmacy); Ms. Ascensión Hernández Encinas (President ASCOL, patient representative); Ms. Mª Teresa Arias Martín (Mental Health Nurse. Member of the Health Care Ethics Committee); Ms. Mª del Carmen Arias de la Fuente (Clinical Trials Management Technician); Ms. Berta Bote Bonaechea (Psychiatry Specialist); Ms. Berta Bote Bonaechea (Psychiatry Specialist); Ms. Mª Teresa Arias Martín (Mental Health Nurse. Member of the Health Care Ethics Committee); Ms. Mª del Carmen Arias de la Fuente (Clinical Trials Management Technician); Ms. Berta Bote Bonaechea (Psychiatry Specialist). Berta Bote Bonaechea (Specialist in Psychiatry); Ms. Ángela Rodríguez Rodríguez (Head of Haematology Unit); Mr. Guzmán Franch Arcas (Specialist in General Surgery and Digestive System); Mr. Antonio Márquez Vera (Physiotherapist); Ms. Ana Martín García (Specialist in Cardiology); Ms. Concepción Rodríguez Barrueco (Primary Care Pharmacist); Mr. Manuel Ángel Gómez Marcos (Primary Care Physician. Head of the Primary Care Research Unit of Salamanca), Ms. Belén Vidriales Vicente (Head of Haematology Section).
